# Supplementary material for: Intraspecies Variation Offers Potential to Improve White Rot Fungi for Increasing Degradability of Lignocellulose for Ruminants
Source: J Fungi (Basel). 2024 Dec 11;10(12):858. doi: 10.3390/jof10120858 (PMC11678441; doi:10.3390/jof10120858)
Supplement: Supplementary file 1 [file jof-10-00858-s001.zip › Supplemental figures S1, S2 and S3.pdf]

Intraspecies Variation Offers Potential to Improve White Rot Fungi for Increasing Degradability of Lignocellulose for Ruminants

Anton S.M. Sonnenberg[1*], Nazri Nayan[1,2], John W. Cone[3] and Arend F. van Peer[1]

1 Plant Breeding, Wageningen University & Research, 6708 PB Wageningen, The Netherlands

2 Department of Animal Science, Faculty of Agriculture, Universiti Putra Malaysia,

Serdang 43400 UPM, Malaysia

3 Animal Nutrition Group, Wageningen University & Research, 6708 WD Wageningen, The Netherlands

* Correspondence: anton.sonnenberg@wur.nl; Tel.: +31-651644640

Supplemental Figures S1, S2,S3

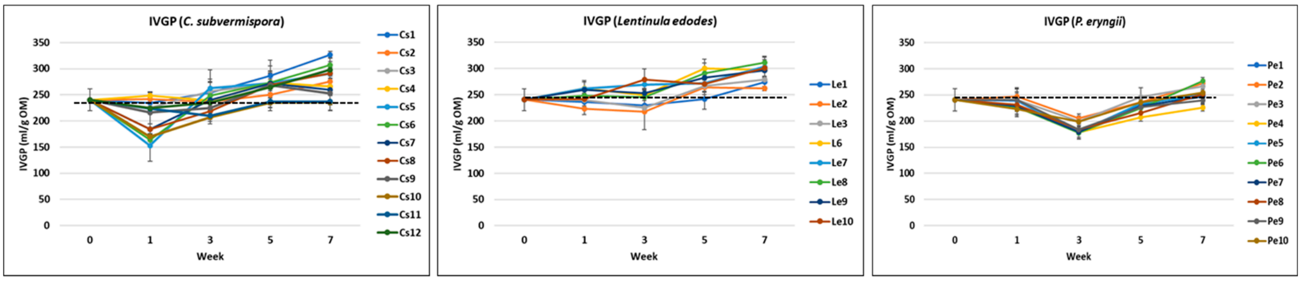

Figure S1. In vitro gas production (IVGP) during 7 weeks of incubation by fugal strains of three different species (*C. subvermispora, L. edodes* and *P. eryngii*).

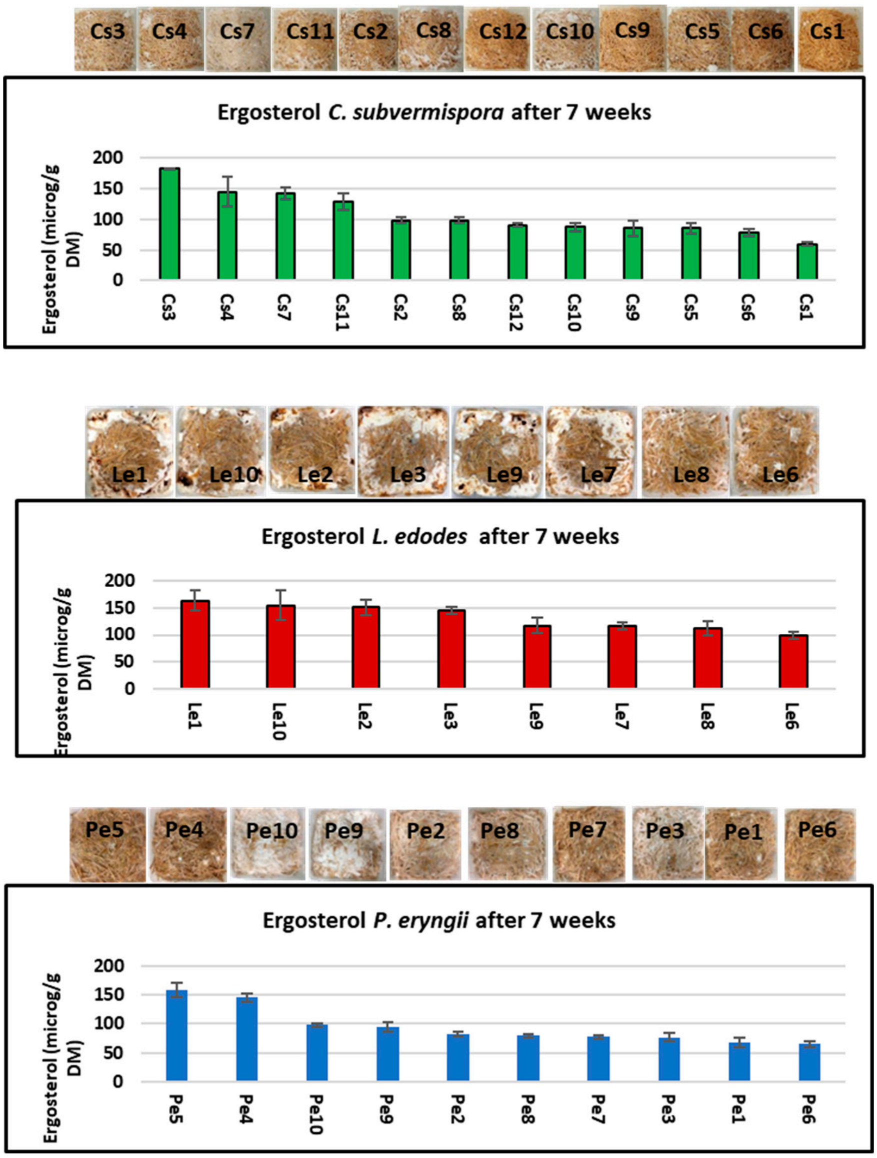

Figure S2. Photographs were taken of the surface of the containers after 7 weeks of fungal incubations (upper parts) showing the whiteness of the substrate as a visual examination of the extent of colonization. Bar graphs show the ergosterol content of each container after 7 weeks of incubation. For the strains of species *C. subvermispora* there is some correlation between the ergosterol content and the whiteness of the colonized substrate, whereas this correlation is not seen for the strains of the species *L. edodes* and *P. eryngii*.

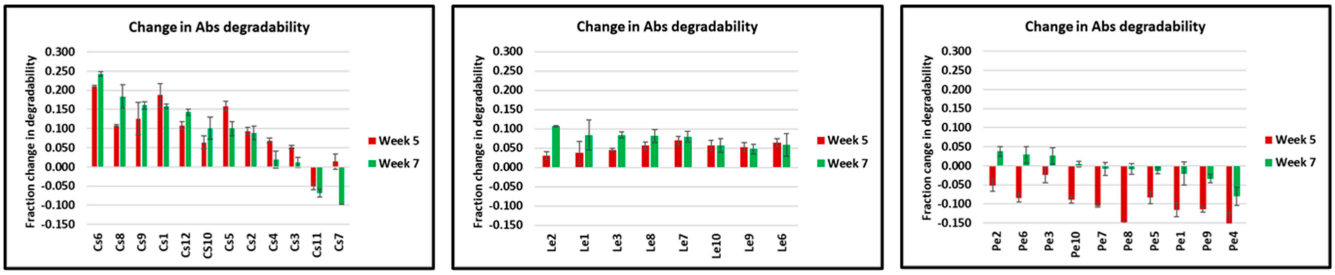

	Change absolute degradability			
Strain	Time (week)	Mean	Pr > F(Model)	Significant
Cs6	7	0.242 a	0.018	Yes
	5	0.210 b		
Cs8	7	0.184 a	0.073	No
	5	0.109 b		
Cs9	7	0.160 a	0.729	No
	5	0.156 b		
Cs1	5	0.170 a	0.118	No
	7	0.158 b		
Cs12	7	0.143 a	0.094	No
	5	0.112 b		
Cs10	7	0.101 a	0.297	No
	5	0.074 b		
Cs5	5	0.164 a	0.022	Yes
	7	0.100 b		
Cs2	7	0.089 a	0.961	No
	5	0.088 b		
Cs4	5	0.070 a	0.064	No
	7	0.019 b		
Cs3	5	0.051 a	0.037	Yes
	7	0.011 b		
Cs11	5	-0.047 a	0.074	No
	7	-0.069 b		
Cs7	5	0.025 a	0.004	Yes
	7	-0.098 b		

Figure S3. Change in absolute amount of degradable organic matter after 5 weeks (red bars) or 7 weeks (green bars) of incubation with fungal strains of three different species. For the species *C. subvermispora* it is clear that for some strains the optimal incubation time is 5 weeks, whereas for other strains this is 7 weeks. For the species *L. edodes* the differences are not significant whereas for *P. eryngii* the amount of absolute degradable organic matter is less for all strains after 5 weeks compared to 7 weeks.
